# Foreign Summary

**Published:** 1839-10-19

**Authors:** 


### FOREIGN SUMMARY.

### Velpeau's Clinical Lectures on Ophthalmia. No. VI.

#### INFLAMMATORY AFFECTIONS OF THE CORNEA.

We are now about to examine a peculiarly interesting class of diseases, which had never been properly studied or described until within the last few years. Until the commencement of the present century, surgical writers, confounding all the inflammatory affections of the eye and its appendages under the name of ophthalmia, made no attempt to ascertain the precise seat of the disease. Far from throwing any light on the nature of the inflammation, they rendered it still more obscure, by adding to the general term ophthalmia the name of various specific diseases, although the inflammatory affections of the eye are not in reality of a specific nature, as I hope satisfactorily to prove to you in the course of these lectures. It is to the prevalence of these doctrines—to the confusion which they engendered—that we may attribute the slight progress which ophthalmology has hitherto made. Pinel and Bichat were among the first to perceive that they have no real foundation. By their labours they proved, in the most satisfactory manner, that in the eye, as in all other regions of the body, the influence which each tissue exercises on the morbid affections of which it is the seat, is such as to render it imperative that they should be studied separately. It is because surgeons were formerly either ignorant of this fundamental principle of pathology, or otherwise neglected to apply it, that we look in vain in their writings for a description of the inflammatory affections of the cornea, considered apart from those of the other membranes of the eyes.

Diseases of the cornea are of extremely frequent occurrence, as will be seen by the perusal of the various statistical accounts which have been published since the attention of practitioners has been directed to the subject. No membrane of the eye, indeed, appears so frequently to be the seat of morbid action. Thus Saunders says, that out of 1942 cases admitted into his infirmary, there were 659 affections of the cornea; more than a third. Out of 250 cases of eye disease which I treated during the two years I was at the hospital of La Pitié, there were 125 cases of disease of the cornea; exactly one-half. Nor can we be surprised at the frequency of these affections, when we reflect on the superficial situation of the cornea, and on the important functions it has to fulfil—functions which are of such a nature that the slightest alteration in its physical properties constitute at once a serious malady. Is it not, therefore, a singular feature in the history of medical science, that keratitis has only been specially studied since the commencement of the present century, and that even since that epoch many ophthalmologists of talent have not examined the disease with the attention which it deserves?

I shall not, in these lectures, treat of all the morbid alterations which may take place in the cornea, but merely study the inflammatory affections of that membrane. Setting aside, therefore, wounds, contusions, tumours, herniæ, &c., I shall forthwith commence the description of keratitis, acute and chronic; and subsequently, as consequences of that affection, I shall describe softening of the cornea, its gangrene, as also the vegetations, abscesses, ulcerations, perforations, and specks, which it so often presents.

*Keratitis.*

Keratitis is the name under which inflammation of the cornea is gerally designated. If we peruse attentively the works of those who have written on ophthalmology, we shall find that several surgeons, in the last century, had a faint view, as it were, of the true nature of the disease, but none described it. Thus Maître Jean, Boerhaave, Deshayes, Gendron, Janin, and several others, merely mentioned the existence of such a malady. It is to Vetch that we owe, in 1807, the first description of keratitis; but he was much surpassed, shortly after, by Mr. Wardrop, whose attention had been for some time directed to this affection. Mr. Wardrop rendered a real service to ophthalmological science by his account of inflammation of the cornea, which I shall take as the basis of what I am about to say; and since that time, keratitis has been admitted iuto all nosological classifications. Since then, also, other ophthalmologists have written on the subject, but in a much more general manner, and their description of the disease is much inferior to that of Mr. Wardrop. This being the case, it is singular that M. Hauffbauer (De Corneâ, ejusque Morbis, &c. Berlin, 1820) should have been looked upon as the first observer of keratitis. M. Mirault, who called the attention of the French medical public to this disease in 1823, although he mentions Saunders and Mr. Travers, does not even allude to Mr. Wardrop. Indeed, many symptoms described by Mr. Wardrop, have been repeatedly brought forward as new.

Keratitis may be either acute or chronic, diffuse or circumscribed, general or partial. It may also be external, interstitial, or deep seated, according as the inflammation attacks the superficial layers of the cornea, the tissue of the cornea itself, or its internal surface. This division, introduced by Mr. Wardrop, has been criticised by some as too minute, and more theoretical than practical. The objection, however, is groundless, for the three forms I have just named may really be met with separately; indeed many of you have repeatedly seen them in my wards. The former and the latter are, it is true, generally combined, the one with conjunctivitis, the other with iritis; but this complication does not do away with the utility of a division which bears directly on the prognosis and on the treatment of the disease. I intend to examine separately each of these forms of keratitis; but before I enter upon such an examination, I must enumerate the causes capable of producing the disease, and also describe the symptoms considered in a general point of view, with which I think it necessary that you should become acquainted as soon as possible. You will, no doubt, soon perceive that many of these symptoms are generally attributed to rheumatic or to scrofulous ophthalmia, to iritis, to choroiditis, &c.

*Causes.*—Keratitis is, as you have already seen, a very common disease, accompanying nearly all the other inflammatory affections of the eye. The causes which may give rise to it are exceedingly numerous, and may be divided into immediate or local and predisposing.

Under the head of immediate causes we may class wounds, blows, burns, foreign bodies brought into contact with the cornea; indeed, every species of external violence, as also various operations practised on the eye. It is thus we see the point of the needle, used in the operation for cataract by couching, wounding the posterior surface of that membrane, occasion inflammation of the membrane. To these causes we may add the existence of any disease of the eyelids or of any other part of the eye; the reflection of the sun in warm countries; the introduction of pus, of virulent matter, or of any other impure fluid between the eyelids.

Exposure of the head or of the body to rain or to damp air, and a sudden change from a warm to a cold atmosphere, have been placed among the causes which are more especially considered to produce what has been called rheumatic keratitis. But this is a form of ophthalmia which, in my opinion, does not exist; and I hope, in a subsequent lecture, to prove fully that my ideas on this subject are correct. These causes may, undoubtedly, give rise to keratitis, but they may also give rise to nearly every other form of malady. We can only look upon them as general causes of disease.

The predisposing causes of keratitis are not well known. In some instances there appears to exist a peculiar predisposition to the affection, independent of hygienic circumstances and constitutional peculiarities, which are, however, generally considered to exercise great influence over the production of the disease. Although, therefore, it is frequently met with in the poorer classes of society—among those who are exposed to the vicissitudes of the atmosphere, whose food is unwholesome and insufficient—you must not suppose that it is only under these circumstances that keratitis makes its appearance. You will see it attack those who live wholesomely, and who are in perfect health: you will meet with it in every age, temperament, and constitution; in the young and in the old—in the lymphatic, the nervous, the sanguineous—in the weak and the robust. It has been said that children are most frequently affected with this malady. Inflammatory affections of the eye are certainly more frequent among them, generally speaking, than among adults, but I cannot say whether the remark applies to keratitis.

Some professions may be considered as predisposing causes. Thus, the reflection of light to which blacksmiths, locksmiths, &c. are exposed, the dust, the corpuscles which float in the air in places where cotton or wool are worked, must often give rise to inflammatory affections of the external tunics of the eye. Watchmakers and jewellers, who make use of magnifying glasses, are also often attacked by this disease.

Many authors assert that climate and season exercise great influence over the frequency of keratitis, and that, consequently, it is much more prevalent in some countries than in others. With respect to seasons, their assertion appears in some degree correct, for although we meet with it in every period of the year, it is certainly more frequent in summer and in winter than in spring and in autumn. I also believe that climate has more or less influence over the disease; but it is extremely difficult to ascertain how far this is the case, as keratitis is not described in other countries under the name by which it is known here. Dr. Mackenzie, in his work on the Pathology of the Eye, does not describe simple keratitis—a fact which rather surprises me. He speaks of rheumatic corneitis, and of scrofulous ophthalmia, but it is difficult to say whether, by these terms, he really means the disease I am now describing. I have, however, heard practitioners say, that they have seen keratitis on the other side of the Channel, and that it was there called rheumatic or scrofulous ophthalmia. I am therefore warranted in concluding that keratitis is as common a disease in London as in Paris.

*Symptoms.*—When the cornea is inflamed, the symptoms are either anatomical or physiological. The anatomical symptoms are those which are supplied by the vascularity of the sclerotica, and by the coloration of the cornea. We will examine them with care, as they will enable you, if properly understood, to recognise keratitis whenever it presents itself to your observation.

*Vascularity of the Sclerotica.—Non-existence of Sclerotitis.*

The vascular appearance which the sclerotica presents in keratitis is one of the most important features of the disease, and differs in many respects from that of the conjunctiva. When the latter membrane is inflamed, it offers numerous vessels of a bluish kind, approaching nearer to the colour of venous than to that of arterial blood, which anastamose freely with one another, and thus constitute a movable vascular net-work. The vascularity of the sclerotica, on the contrary, is constituted by exceedingly minute capillary vessels, which persons, unaccustomed to the observation of eye-diseases, would at first with difficulty distinguish. Their colour is not always the same: with some persons they are of a vivid red; with others, especially in the first stage of the disease, they are of a pale carmine colour. These vessels advance in a parallel manner, without visibly anastamosing with one another, and form a kind of radiated vascular zone round the cornea. They seem deeply imbedded in the tissue of the sclerotica, and, on arriving at a short distance from the cornea, cease to be visible. They do not all disappear in the same manner; some, leaving the sclerotica, seem to continue their course in the conjunctiva; others appear to re-enter the eye; whilst others remain in the sclerotica. You will easily understand this distribution of the vessels which constitute the vascular zone I am describing, if you remember what has been already said regarding the distribution of the arteries of the eye. Near the cornea, the vascularity assumes the appearance of a narrow band or ring, which may be complete or incomplete, regular or irregular. This circular band advances about half a line on the cornea, in the same manner as the rim of a watch advances over a watch glass. I cannot say whether it adheres or not to the cornea, as I have never had the courage to ascertain the fact by attempting to introduce a foreign body between them. I am inclined to think it is a fold formed by the injected vessels of the sclerotica. However we may account for its formation, it cannot be confounded with chemosis, which we have seen accompanying inflammation of the conjunctiva. It is merely a thin, narrow, membranous band, neither red nor tumefied, presenting, indeed, none of the characters peculiar to chemosis. In some cases, the membranous band does not exist; the vessels which constitute the vascularity pass on to the cornea, and may be traced, when it retains its transparency, under the form of exceedingly minute filaments, until they terminate about a quarter of a line, or half a line, from its circumference. Sometimes we find neither vascular band nor minute vessels extending into the tissue of the cornea, but a gray circle, about a quarter of a line in width, which either entirely surrounds the cornea, or merely borders it opposite the inner and outer angle of the eye. This is what some authors have improperly called the arthritic circle; it may be assimilated to the senile circle in the healthy eye.

The vascularity of the sclerotica does not only differ from that of the conjunctiva by the distribution of its vessels, but there are also other characters which may serve to distinguish them. Thus, the injection of the sclerotica diminishes as we recede from the cornea; that of the conjunctiva, on the contrary, increases. The width of the vascular zone of the sclerotica, although it varies according to the acuity of the inflammation, rarely exceeds two or three lines; whereas the redness of the conjunctiva is, generally speaking, universal. On pressing the inflamed conjunctiva, and drawing it slightly aside, through the medium of the eyelids, you will find that it is extremely moveable, and that the pressure, expelling the blood from the vessels, renders it much paler. This is not the case with the vessels of the sclerotica; they remain immoveable; no change takes place in their relation to one another, or to the cornea; nor does their colour undergo the least alteration.

Such are the characters by which the vascularity of the sclerotica may be recognised. The interpretation which I give to this phenomenon is very different to that which is generally adopted by ophthalmologists. I consider it as indicating the presence of keratitis or iritis, whilst they look upon it as symptomatic either of a specific inflammation, or of sclerotitis. Thus, in the work of Dr. Mackenzie—one of the most able writers on ophthalmology of the present day—the vascular zone of the sclerotica is described as the characteristic indication of sclerotitis, a disease the very existence of which I consider improbable. As I wish to prove to you that the opinions I profess are correct, I shall take this opportunity to lay before you the reasons which have induced me to question the existence of a disease so universally recognised and described.

Real sclerotitis is, in my opinion, an extremely rare disease, if even it has ever been really observed. I do not mean to deny the possibility of the sclerotica becoming inflamed; I am far from pretending to assign a limit to inflammation; but I am firmly convinced that the vascular zone, as also the other symptoms which are described by authors as indicating the presence of sclerotitis, are merely the result of an inflammatory affection of the cornea, or of the iris. This opinion may at first appear paradoxical; I hope, however, soon to place it in a more favourable light. Let us analyze with care the symptoms which authors attribute to this malady; we shall then see whether the lesion of some other membrane of the eye might not equally well account for their presence—whether, indeed, some of these symptoms are not altogether inapplicable to the affection to which they are referred. We must also, in pursuing this investigation, take into consideration the anatomical texture of the sclerotica, and the nature of the inflammatory affections which attack similar tissues in other parts of the body. We shall thus be able to judge how far the German ophthalmologists are right in giving to this supposed affection the name of rheumatic ophthalmia: this they have done from preconceived theoretical notions. Rheumatism attacks fibrous tissues; and the sclerotica being fibrous, they have concluded that the inflammation of its tissue, which they suppose to be indicated by the vascular zone it so often presents, is of an arthritic nature. Even Dr. Mackenzie, however, although under the influence of the school of Beer, is obliged to admit that he has often met with this symptom in patients who had never been affected with rheumatism in any shape; but we will, for the moment, lay aside all theoretical ideas, in order to submit the facts on which the existence of sclerotitis is founded, to an accurate analysis.

The first question which presents itself to us is, whether vascularity of the sclerotica may be attributed to any other cause than to inflammation of that membrane? To this I shall answer decidedly, that it may; that it is quite as rational to attribute this vascularity to keratitis or iritis, as to inflammation of the sclerotica; and in this I am sure you will agree with me, if you remember what I have said respecting the distribution of the vessels of the eye. When the cornea or the iris are inflamed, their vessels become injected; and, owing to the numerous anastomoses which exist between the vascular system of these organs and that of the sclerotica, the vessels of the latter membrane also become turgid. The redness of the sclerotica is not, therefore, to be attributed to inflammation, but to the injection of the vessels which exist in its tissue. If, indeed, you examine the eye very attentively, you will find that the sclerotica remains perfectly white between the vessels. An experiment which I have often made, satisfactorily proves that vascularity of the sclerotica is not dependent on inflammation of that organ. I have repeatedly endeavoured, by wounding the sclerotica, to give rise to the vascular zone, which is generally looked upon as the symptom of its inflammation, but always without success. A speck, an ecchymosis, or a slight purulent affection, were the only consequences of the lesion. When, on the contrary, I have wounded the cornea, I have generally produced the vascularity of the sclerotica. I do not, however, consider these arguments alone of sufficient weight to warrant our denying the existence of sclerotitis, as we must also allow that if the tunica albuginea were to become inflamed, that inflammation would be as likely to give rise to vascularity as either keratitis or iritis. We will, therefore, rest satisfied, for the present, with the knowledge that this phenomenon may be explained in a satisfactory manner, without our being obliged to suppose an inflammatory affection of the sclerotica.

But if we continue our analysis of the symptoms, we shall find that there are two which cannot be allowed to depend on an affection of this membrane—I allude to intolerance of light, and to effusion of tears. These symptoms are very generally ascribed to the imaginary disease we are now examining, although a few moments' reflection must convince you that they could not possibly be occasioned by such an affection. The sclerotica is merely an organ of protection, and has nothing to do with the visual functions, nor has it any connexion with the secretion of tears. I shall not, however, enter at present into any details on the subject, as I shall have to treat of these phenomena in a future lecture; I will then prove clearly to you that they are characteristics of keratitis, retinitis, or iritis.

The examination of the symptoms which sclerotitis is said to present, is evidently far from conclusive with regard to its existence. Let us now see if theory will be more favourable to the general opinion.

If the symptoms which authors ascribe to sclerotitis really belong to an inflammatory affection of that membrane, it must be considered one of the most common of the various forms of ophthalmia; indeed, those of you who follow my practice attentively, will remember having seen these symptoms in at least half the patients we have received in the wards for diseases of the eye this year. Now, when we reflect on the anatomical structure of the sclerotica—when we consider that it is a tissue which, in other parts of the body, is so rarely inflamed, that some pathologists entertain doubts as to its ever being the seat of inflammatory action—how can we possibly reconcile our minds to the extreme frequency of sclerotitis? Again, the inflammation of a fibro-cellular membrane no more remains confined to one spot, limited to a circular portion of the organ, than that of a mucous or a serous membrane, but spreads in every direction. The vascular zone of the sclerotica, on the contrary, never extends to the posterior portion of the membrane; it merely encircles the cornea, gradually disappearing as it recedes from it. You are also, no doubt, well aware that when tissues analogous to the sclerotica have been inflamed, there generally remains some trace of the inflammation, such as thickening, &c. Has any similar lesion ever been seen on the sclerotica, when once the ocular inflammation is entirely subdued?

You see, then, that both observation and reasoning are equally contrary to the existence of sclerotitis; I therefore feel myself perfectly justified in excluding it from the list of ophthalmiæ, until, at least, its existence shall have been demonstrated in a much more satisfactory manner than has hitherto been done.

### FOREIGN HOSPITAL PRACTICE.

#### LA CHARITÉ.

*Fall from a Height.—Inflammation of the Spinal Marrow.*

*June* 6.—A locksmith, aged twenty-three, was admitted to-day. Three days ago he fell from the second story, in consequence of his stepping upon a board which was inadequately supported. He remained unconscious for about ten minutes; afterwards he was able, although feeling rather stupified, to return to his work. There is a slight wound of the scalp in front. The next day he felt great pain and stiffness in the neck, and these have been increasing up to the present time. He supports his head with his two hands, and seems unwilling to try to hold it up without their assistance; he was bled to sixteen ounces.

In the course of the night he was delirious, but is quite tranquil and rational this morning; pulse quick, but not full or strong. Just above the spinous process of the seventh cervical vertebra he complains of much tenderness; here there is an evident depression, more, in M. Velpeau's opinion, than should naturally exist. One of the students said that he could feel a crepitation, but this M. Velpeau could not distinguish. There appears to be an evident degree of increased mobility in the neck at this part. There is no displacement of the vertebræ observable by placing the finger in the pharynx. From all the symptoms M. Velpeau is inclined to diagnosticate a fracture of some of the lower cervical vertebræ. There is no paralysis of any of the extremities. The treatment is to bandage the head, so that its movements may be restricted, and to leech behind the ears, to prevent, if possible, a return of his delirium.

7. Much the same.

8. Was delirious all yesterday, and during the night, and is much more so this morning, on account of the excitement attendant upon the visit. The delirium is of a mild character. His skin is of moderate temperature, and his pulse is neither strong nor very frequent; he is, however, obliged to be restrained with a strait-waistcoat.—To have calomel.

9. Much the same.

10. Died in the middle of the night.

*Appearances after Death.*

No fracture of the cervical vertebræ, but diffused suppuration over the entire surface of the spinal marrow, and underneath the dura mater. This layer of pus was semi-fluid, and thickest near the cervical region. In various points of the surface of the brain and cerebellum there are different spots of purulent deposition underneath the arachnoid membrane.

It is remarkable in this case that latterly the head and neck were maintained in a curved direction, backwards, and that there was no paralysis of the extremities.

M. Velpeau has seen several cases of this kind, and in all of them he observed this tendency to support the head with the hands, and, in his opinion, when he finds this symptom present, after any injury to the head or neck, as a blow, fall, &c., he is convinced that the cervical portion of the spinal chord has suffered severely.

*Necrosis of the Upper Third of the Humerus.—Operation.*

H. P., aged nineteen, comes from the country. Two years ago he suffered from abscesses over the upper part of the femur, which, bursting, gave exit to some pieces of necrosed bone. After this was healed, the right arm became similarly affected; an abscess formed in the upper part was opened, and discharged a quantity of pus. The upper part of the humerus is very much enlarged by the formation of new bone. The probe discovers dead bone within the new osseous sheath. Since his entrance into the hospital he has suffered from phlegmonous erysipelas, followed by destruction of a considerable portion of cellular tissue.

*June* 21.—To-day the dead bone was removed by the following operation:—The youth having been laid upon the table, the surgeon made a curved incision in the deltoid muscle down to the bone. The convexity of the curve was directed backwards, and its extent might be about three or four inches. This flap was dissected off the bone and reflected inwards; one small vessel on the outer side was ligatured. The new osseous case was opened with Hey's saw, and the mallet and chisel, and two portions of the entire shaft of the humerus were removed from the cavity, about two inches in extent. The boy complained very much when the pieces of dead bone were being torn out of the cavity of the new bony case. There was considerable oozing of blood from the vascular tissues of the periosteum and medullary membrane.

24. Wound dressed again to-day. The flaps, which had been reflected upon the charpie, were again everted, so that the charpie should be extracted and fresh portions renewed, when they were again replaced. The boy complains bitterly when the medullary cavity is touched; he is doing as well as can be expected.

29. Surface of the new bone and wound granulating freely. Boy in good health.

*July* 2.—Edges of the wound are being approximated with slips of adhesive plaster.

15. Wound is looking very well.

19. Doing very well.

#### HOTEL DIEU.

*Dislocation of the Humerus forwards—Reduction.*

*Case* 1.—June 20. A young man, aged twentythree, dislocated the left humerus three days ago, by falling, when attempting to push a heavy cask upon another. Does not know how his arm might be placed at the time of its occurrence, but he fell down, slipping his footing. There is tumefaction under the pectoral muscle, and the head of the bone is to be felt there, both by the fingers introduced into the axilla, and underneath the muscle. The elbow is widely separated from the side. The arm is shorter than the other by half an inch. Reduction was easily effected by the method of White, viz., by placing the humerus in a vertical position, and using it as a lever over the acromion process. The reduction was not accompanied by any noise, but was recognisable by all the symptoms being removed.

M. Roux says that there is a pathological distinction between this kind of dislocation of the shoulder-joint, when it is primitive or secondary. Thus, in the primitive form, the head of the bone lies between the subscapularis muscle, and the venter of the scapula; whereas, in the secondary form, the head of the bone lies upon the muscle, and between it and the pectoralis.

*Case* 2.—(La Charité.)—July 2. A labouring woman, age forty-one, four days ago fell as she was crossing a yard, and dislccated the left shoulder. She does not know in what manner she fell, as she was suddenly seized with a giddiness and stupor to which she is subject. Pain in the part. The head of the bone may be felt under the coracoid process, and with the fingers in the axilla. The limb is very little shorter than the other. The subclavicular fossa has nearly disappeared, while the subacromial is greatly increased. The deltoid muscle is stretched a little. She can raise the hand to the forehead, but with great difficulty.

The arm was easily reduced by making extension from the trunk in a horizontal direction. The bone returned into the socket with a snap.

---

*June* 15.—To-day M. Roux removed a large lobulated steatoma from the soft palate, of the size of a hen's egg. The operation was very simple, and consisted in laying open the mucous membrane and exposing the tumour, and then tearing it out with the fingers.

---

*M. Roux's Experience in Fissured Palate.*

Previous to the introduction of a patient into the theatre, who was to undergo the operation of staphyloraphy, M. Roux said that he had been successful in these cases in the proportion of three to four, when there was no fissure of the hard palate; whereas, when this last deficiency was present, as well as a fissure in the soft palate, his success was diminished to one in four. The case which he had intended to operate upon was one in which the operation of hare-lip had been long since performed. He was obliged to give up his intention, as he could not get at the parts with ease, on account of the difficulty which the young man had in opening widely his mouth, and also his not being able to maintain his tongue in the required position.—*Lancet.*

---

*Paralysis of the Facial Nerve in Infants, produced by the Forceps.*—Several years ago Professor Paul Dubois first distinguished this singular affection, which has been pretty fully described in a recent thesis by M. Landousy. Paralysis of the eyelid during sleep, and of the nostril, during excited respiration, with distortion of the features during crying, are the natural results of an injury done to the facial nerve.—*Ib.*

---

*Daguerre's Apparatus of Photography.*—The process of photographic drawing was publicly exhibited by M. Daguerre, by order of the Minister of the Interior, on the Quai D'Orsay, Paris, on the 7th of September. The following account of the operation, as performed on that occasion, from the *Journal des Débats*, more satisfactorily describes the apparatus and process than any previous one that we have seen:

M. Daguerre took a plate of copper plated with silver, (not silvered merely,) and rubbed the silver surface slightly with sweet oil and very fine pumice-powder, making use of little balls of cotton wool. The object of this operation is to cleanse and dull the surface completely; and, in performing it, it is necessary to rub first with a circular motion, and afterwards in straight lines. That done, he washed the plate with a solution of nitric acid, composed of sixteen parts of distilled water, and one of acid: the plate was then slightly warmed by passing it over the flame of a lamp, with the silver surface uppermost; and the washing with diluted nitric acid, as before, was repeated. It was now ready to receive the coating of iodine, which was thus applied. The light being excluded by closing the shutters of the apartment, the plate, fixed on a small board, was placed, with the silver surface downwards, over an aperture the size of the picture required, in the lid of a box, at the bottom of which was the iodine; half way in the depth of the box was a wooden frame on which a piece of muslin was strained; as the iodine evaporated, the fumes rose through the muslin, and were thus diffused equally over the surface of the plate, forming on the silver a coating of iodide of silver, the colour of brass.

The plate, thus prepared, was placed in the camera obscura—the focus having been previously adjusted by trying the effect of the picture on a piece of ground glass; and it was suffered to remain till the action of the sun's rays on its surface was considered sufficient. The time required varies from seven to forty minutes, according to the season and the state of the weather; and, as not the slightest trace of the effects of light is visible on the plate, the judgment of the operator, derived from experience, is his only guide. In withdrawing the plate, great care must be taken that no light whatever falls on it, previous to the operation that follows. The plate was then fixed at an angle of forty-five degrees, in a box, at the bottom of which were placed about two pounds of mercury in an earthen pan: the mercury was heated to sixty-five centigrade, (about one hundred and seventeen degrees of Fahrenheit,) by means of a lamp beneath the pan; and the globules of mercury rising combined with the surface of the plate, till in a few minutes the picture appeared. This operation was watched through a piece of glass inserted in front of the box; and, the moment it was completed, the plate was taken out and washed with distilled water saturated with common salt, or with hyposulphite of soda, heated to a degree below the boiling point: the process was then completed.

M. Daguerre observed, for the information of those who wished to make trial of the process, that, after the iodide of silver has formed, the plate, if kept from the action of light, can remain for an hour without risk of danger; and that, after the view is transmitted as above, it can remain excluded from light for four hours without injury, before submitting it to the operation of the vapour of mercury.

---

*Particulars of the Disease and Post-Mortem Examination of a Lithophagus.* By Dr. Neret, Physician to the Hospital St. Charles, at Nancy.—Dominique Henrion was born at Metz, in 1761. Being but little pleased with the occupations to which he was put when young, he began, at the age of twenty-two, to swallow pebbles. Sometimes he took them whole, and without any preparation; sometimes he broke them between his teeth, after having reddened them in the fire, and then suddenly plunged them in cold water. By this means he passed himself off as a savage from America. (!)

Several years since, he had fixed his abode at Nancy, and there continued the same kind of life, swallowing every day a greater or less number of stones, which on some occasions amounted to thirty or forty. The largest were of the size of a large nut, but they were commonly smaller, and Henrion used to demonstrate their presence in his stomach by the noise they made when he struck his epigastric region. He passed them by stool in twenty-four hours after taking them, and often made use of them again next day. He swallowed also live mice, but only one a day, as well as moderate-sized crabs, after having cut off their claws. As to the mice, when they were put into his mouth, they at once rushed into the pharynx, when they were soon suffocated, and the deglutition of them was then facilitated by that of a stone. The next day they passed from the rectum, with their skins destroyed, and covered with mucus.

This man continued the same kind of life up to the 1st of April, 1820. At that time, after having filled his stomach with a considerable number of stones, he swallowed, for a small sum, a tinned iron spoon, five inches and a half long, and an inch broad. Some hours after, he was seized with vomiting, which continued to the time of his death. He first vomited bilious substances, and then fluid tinged with blood, and of a very fetid smell.

In this state he was brought to the hospital on the 6th of April. The vomiting continued; the thirst was excessive; the pulse small and irregular; the abdomen hard, distended, and painful, especially beneath the umbilicus. Next day, after an enema, the patient passed five stones by stool, but he grew worse, and died on the following day, aged fifty-nine.

At the autopsy, the medullary substance of the brain was very firm; there was some serum in the ventricles, and some hydatiform vesicles on the choroid plexuses. The lungs were firmly adherent to the walls of the chest, but healthy. The heart was in no respect remarkable. On opening the abdomen, the stomach was found distended with gas; the great omentum was inflamed, and there were points of suppuration on its anterior surface, as well as on the transverse arch of the colon. The whole internal surface of the digestive tube and the parietal peritoneum participated in the inflammation. On raising the great omentum, an aqueous fluid flowed out, in which there flowed out globules of oil, which appeared to be the medicine that the patient had taken, and which indicated a perforation of the intestinal canal. In fact, the spoon could be felt in the transverse part of the duodenum, and it was seen that the handle, after having pierced this intestine where it curves to take the name of jejunum, was sticking out two inches beyond it. Internally, the duodenum was covered by a thick layer of greyish mucus.

The internal surface of the stomach was red and inflamed; here and there were points of ulceration; the pyloric orifice was more than usually dilated; but the degree of dilatation of the pharynx, œsophagus, cardia, and the stomach itself, was by no means remarkable. There were thirty-two pebbles in the great cul-de-sac of the stomach, with liquid and oily fluids; some stones were also found in the duodenum, and in some other parts of the intestinal canal; the cœcum contained five; so that the whole number found in the course of the digestive tube was fifty-three, and they weighed altogether one pound and three ounces: the largest weighed five drachms. In form they differed considerably: there were some long and sharp, and others which approached the cubic form; but in general they did not appear to have been chosen to prevent the wounding of the digestive canal.

The liver was large and hard, and the gall-bladder contained a small quantity of fluid.

This case was published soon after its occurrence by Dr. Neret's predecessor; but as the details then given were insufficient, and Dr. N. has himself had the opportunity of examining the case, he thought it but right again to print it, and to add that which was wanting in the previous account.—*London Medical Gazette, from Archives Générales de Médecine*, Juillet, 1839.

---

*Citrate of Quinia in Intermittents.*—This is recommended by Professor Beraudi, because it is equal in effect to the sulphate, in doses one-third smaller; and because it is more easily borne by the stomach, and causes less congestion in the brain.—*Ib.*, *from Bulletin de Thér*, and *Schmidt's Jahrb.*

*Petechial Fever at St. Petersburgh.*—A correspondent of the *Zeitschrift für die gesammte Medicin*, for August, 1839, who dates from Petersburgh, says, petechial typhus fever has been really epidemic among us this year. It was not only the hospitals which were full of patients who belonged to the working classes suffering under this malady, but an extraordinary number of cases occurred among opulent families. It was suprising that the patients never seemed to be delirious during the course of the disease, but went on speaking and acting with consistency; yet when the disease was over, towards the end of the third or fourth week, they awoke with astonishment into a consciousness of their condition. The reason probably was, that the typhus action was almost entirely spent upon the lungs and skin, so that the reaction amounted to inflammation in these organs alone, while the brain remained continuously narcotized. With us, as in Germany, the measles have been of an unfavourable character this year, from thorax, and even with irritation of the membranes of the brain; they also attacked many persons for the second and even the third time.—*Lon. Med. Gaz.*

*Syrup of Copaiba.*—Mouchon recommends a syrup as a good form of giving copaiba. Four ounces of the purest copaiba are to be rubbed in a marble mortar with thirty-two grains of calcined magnesia, till perfect union takes place; sixty-four drops of oil of peppermint and sixty-two ounces of simple syrup are then to be added with continual stirring, until a homogeneous syrup is produced, which is to stand for twenty-four hours, and then be poured into bottles. Or, four ounces of the balsam may be made into an emulsion, with two ounces of gum arabic in two ounces of water; and this may then be mixed with the oil of peppermint and the simple syrup.—*Ib.*, *from Journ. de Pharm. du Midi*, and *Schmidt's Jahrb.*

*On the insufflation of Mercurial Powder in the treatment of Excoriations of the Neck of the Uterus.* By M. TROUSSEAU.—M. Trousseau reports a case of simple excoriation of the neck of the uterus, unsuccessfully treated by the super-nitrate of mercury, and which was rapidly cured by the insufflation of mercurial powder. This treatment has since succeeded, in the hands of the editor, in a large number of cases of superficial erosion or ulceration, (*not cancerous*,) of the neck of the womb. The following is the plan employed:

A powder is formed, by taking of the Proto-Chloride of Mercury, (obtained from precipitation,) gr. xviij.

Of the Deutoxide of Mercury gr. xviij.

Of powdered sugar ℥j.

To be carefully mixed and preserved for use in a flask or box.

He introduces an ordinary speculum; with a large pair of uterine forceps, inclosing some charpie or carded cotton, the surface of the cervix is wiped, and all the mucus covering the membrane removed. Then, he places in one of the extremities of a glass tube, a foot long, from four to twelve grains of mercurial powder, which he insufflates upon the diseased part. The speculum is then withdrawn; the powder remains applied to the cervix, and is soon partially dissolved. This simple operation is repeated two or three times a week; afterwards only once in five or six days,—then every eight, or every fifteen days, till a complete cure takes place. In the lady, whose case is reported by M. Trousseau, the cure was completed in two months.—*Journal des Connaissances Medico-Chirurgicales.*

*Autoplasty after the Excision of Cancerous Tumours.*—M. Phillips, a surgeon at Liege in Belgium, has addressed a letter to Professor Diffenbach, of Berlin, with the view of showing "what important changes diseased tissues frequently undergo by being brought into contact with healthy ones, and how this important therapeutic principle may be taken advantage of, for the purpose of preventing the return of cancerous and other malignant tumours in parts from which such tumours have been extirpated."

The Professor himself has alluded, in one of his published letters, to the practical application of the above principle; and in 1835, Dr. Martinet de la Creuse published in the Gazette Medicale, a memoir on autoplasty as a therapeutic or curative means, and narrated at the same time the particulars of four cases in illustration: two of these were examples of cancer in the face, and, in the other two, the mamma was the seat of the disease.

The attention of M. Phillips had been much excited by this paper, and he therefore resolved to test the advantages of the practice for himself. His first experiments were performed on dogs; but as we cannot well admit that the tumours in these animals, which M. Phillips designates as cancerous, were really of a malignant nature, it is quite unnecessary to detail them. We shall, therefore, only allude to the results of certain operations on the human subject.

Case 1. *Excision of a Cancerous Mamma; Return of the Disease; Second Excision, and Transposition of a Flap of Healthy Skin to fill up the Wound.*—Mad. D——, 54 years of age, submitted to the extirpation of the left mamma, which was affected with an open cancer. As a large portion of the integument required to be removed, the edges could not be brought together, and the wound was therefore allowed to suppurate. *This*, says M. Phillips, *was a great error which I committed.** A month after the operation, the wound was entirely cicatrized. Within a twelvemonth,

* M. Phillips seems to recommend the practice, so much followed by Dieffenbach, of making one or two lateral incisions in the adjacent skin, to permit the extension and approximation of the edges of the wound.

however, a fresh tumour, which was evidently of a malignant character, made its appearance in the site of the cicatrix. M. Phillips resolved, after excising this new tumour, to transplant a portion of healthy integument to fill up the wound. He therefore dissected a flap, of the dimensions required, from off the adjoining integuments, and transported it to the place which the tumour had occupied, (*le lambeau, que je devais emprunter sur un coté de la poitrine, fut deplacé et transporté sur le lieu où la tumeur avait vecu;*) it was secured in its place by fourteen points of suture.* The wound was then dressed with compresses wetted with cold water.

Next day one of the points of suture was removed, for the purpose of giving discharge to some fluid in the wound; and in two days afterwards all the other stitches were withdrawn. Already the flap was found to be adhering in all its extent; the immediate union was quite complete, and unattended with any suppuration. The cancer has not returned. (The date of the operation is not given.—*Rev.*)

*Case 2. Cancer of the lower Lip; Excision of the Tumour, and Transplantation of a portion of Healthy Skin; Cure.*—Although the greater portion of the lip was involved in the morbid degeneration, its mucous lining was found to be quite exempt from the disease. M. Phillips therefore determined to save this, by dissecting from off it the indurated integuments and muscular substance, and leaving it for the purpose of adhering to the new flap which he proposed to *graft* upon the spot.

This flap was obtained from the integuments under the jaw, and, being made to correspond with the size of the wound of the lip, was fixed in its new place by means of several sutures, the mucous lining being brought or turned over its upper edge, and secured in its position by six points of the interrupted suture.

On the fifth day after the operation, the stitches were removed, and it was then found that the flap had adhered at every point, except where its pedicle was attached.

The wound under the chin, from which the flap had been removed, was healed at the end of the third week.

M. Phillips, in the treatment of this, as well as of the preceding case, had employed very active antiphlogistic means after the operation. The flap had become tumefied, red, and shining, as if it had been coated with varnish; M. Phillips scarified it in a number of places, and then applied cold wetted compresses to it.

In another case, in which the operation of rhinoplasty was performed, the new nose remained, for two hours after the operation, quite pale, cold and insensible; the application, however, of iced water was still continued, and the colour and warmth of the flap began gradually to return. At the end of six hours, it had become considerably swollen, and heated; a great number of leeches was immediately applied, and the oozing from the bites was encouraged. Next day, a good many scarifications were made with the lancet, and these were repeated *de deux en deux heures.* In the afternoon, the patient was bled freely from the arm, and the cold water was kept constantly applied to the head and face. On the third day, upwards of thirty of the stitches (forty had been used) were withdrawn, and the new nose was found every where adhering.

A smart attack of erysipelas of the face came on at this time; but fortunately the re-adoption of very active antiphlogistic means rapidly arrested its course, and eventually the patient *jouit de tous les avantages d'un organe dont il etait privé depuis dix années.—Medico-Chirurg. Rev., from Bulletin Medical Belge.*

* We should not omit to state that the bleeding from the divided vessels had been arrested by torsion, and that no ligatures had been applied.

---

*Good method of administering Iron.*—Dr. Meurer, of Dresden, has long been in the habit of recommending to his patients the following method of taking a chalybeate in an effervescing draught:

℞. Ferri sulph. cryst. ʒss.

Sacchari albi ʒj.

Misce et divide in pulv. xij.

℞. Natri carbon ʒss.

Sacchari albi ʒj.

Misce et divide in pulv. xij.

One powder from each packet is to be dissolved separately, and the solutions mixed together. About a grain or so of the fresh precipitated subcarbonate of iron, mixed with a small proportion of sulphate and of carbonate of soda, is thus taken in an agreeable and useful form.—*Caspar's Wochenscrift.*

There is a well known formula for steel pills, which have acquired a high celebrity in Paris for the cure of chlorosis and its dependent affections. They often pass by the name of *Bland's pills.* M. Bland, the chief physician of the hospital de Beaucaire, having first introduced them to the notice of his countrymen. He has recently published an article in the *Revue Medicale,* wherein he has adduced a multitude of cases illustrative of the curative quality of his pills.

They are to be prepared as follows:

Take of gum *adragante* (tragacanth?) six grains, and a drachm of water, so as to form a thick mucilage. To this mucilage add half an ounce of fresh pulverized sulphate of iron, and when this is perfectly and uniformly mixed, add the same quantity of subcarbonate of potash, and blend all well together, so as to form an even mass. The mass acquires at first a yellowish-green colour, and then becomes of a deeper green, acquiring at the same time a soft consistence. The mass is to be divided into forty-eight pills, of which from one to three are to be taken daily.

M. Bland states that, if his directions are faithfully followed in the preparation of the pills, the mass retains for several days a soft consistence: it may, however, be divided into pills, either early or late, as the physician may require.—*Medico-Chirurgical Review.*

